# Suppressive Interaction Approach for Masking Stale Note of Instant Ripened Pu-Erh Tea Products

**DOI:** 10.3390/molecules24244473

**Published:** 2019-12-06

**Authors:** Ting Zhang, Hui Ni, Xu-Jian Qiu, Ting Li, Liang-Zhen Zhang, Li-Jun Li, Ze-Dong Jiang, Qing-Biao Li, Feng Chen, Fu-Ping Zheng

**Affiliations:** 1 Beijing Advanced Innovation Center for Food Nutrition and Human Health, Beijing Technology and Business University, Beijing 100048, China; yczkting@126.com (T.Z.); chenfeng@jmu.edu.cn (F.C.); 2 College of Food and Biological Engineering, Jimei University, Xiamen 361021, China; xjqiu@jmu.edu.cn (X.-J.Q.); xmtingli@163.com (T.L.); xmliangzhen@126.com (L.-Z.Z.); ljli@jmu.edu.cn (L.-J.L.); zdjiang@jmu.edu.cn (Z.-D.J.); qbli@jmu.edu.cn (Q.-B.L.); 3 Key Laboratory of Food Microbiology and Enzyme Engineering Technology of Fujian Province, Xiamen 361021, China; 4 Research Center of Food Biotechnology of Xiamen City, Xiamen 361021, China; 5 Department of Food, Nutrition and Packaging Sciences, Clemson University, Clemson, SC 29634, USA

**Keywords:** instant ripened Pu-erh tea, stale note, sensory evaluation, suppressive interaction, masking

## Abstract

The unpleasant stale note is a negative factor hindering the consumption of instant ripened Pu-erh tea products. This study focused on investigating volatile chemicals in instant ripened Pu-erh tea that could mask the stale note via sensory evaluation, gas chromatography-mass spectrometry (GC-MS), and gas chromatography-olfactometry (GC-O) analyses. GC-MS and GC-O analyses showed that linalool, linalool oxides, *trans*-β-ionone, benzeneacetaldehyde, and methoxybenzenes were the major aroma contributors to the simultaneous distillation and extraction (SDE) extract of instant ripened Pu-erh tea. Sensory evaluation showed that the SDE extract had a strong stale note, which was due to methoxybenzenes. By investigating suppressive interaction among flavour components, the stale note from methoxybenzenes was shown to have reciprocal masking interactions with sweet, floral, and green notes. Moreover, the validation experiment showed that the addition of 40 μg/mL of *trans*-β-ionone in the instant ripened Pu-erh tea completely masked the stale note and improved the overall aromatic acceptance. These results elucidate the volatile chemicals that could mask the stale note of instant ripened Pu-erh tea products, which might help to develop high quality products made from instant ripened Pu-erh tea.

## 1. Introduction

Teas have various health benefits and are widely consumed over the world [1]. China is the largest producer of tea in the world, contributing 36% to the total global production, followed by India (21.2%), Kenya (7.8%), Sri Lanka (7.0%), Turkey (4.8%), Vietnam (4.6%), and Iran (3.3%) [2]. Pu-erh tea is a kind of post-fermented tea, originally produced in the Yunnan province of China. According to the statistics of Chinese tea market, the output of Pu-erh tea is about 116,500 tons and the comprehensive market value is 25.5 billion RMB in 2018. It has gained more and more popularity and attracted much attention especially in China and some other Asian countries for its potential health function, including antioxidant activity [3], preventing cancer [4], inhibiting cholesterol biosynthesis [5] and antimutagenic, and antimicrobial activities [6]. Recently, with the increasing market of ready to drink/eat products, more and more Pu-erh tea is being processed to instant tea products. Although instant tea is prepared via water extraction, vacuum/membrane concentration, and spray/freeze-drying [7], instant Pu-erh tea has a strong stale note inherent from ripened Pu-erh tea due to methoxybenzenes. Some consumers are used to accept the stale note; however, the majority of the populations feel that the stale note is undesirable, which negatively affects the aroma of the drink/beverages made from instant ripened Pu-erh tea.

Pu-erh tea has been determined to have 61 and 67 volatiles via GC-MS analysis by the aids of simultaneous distillation extraction (SDE) and solid phase micro-extraction (SPME), respectively [8]. Among them, methoxybenzenes that are synthesized from tea catechins through microbial degradation and methylation during the post-fermentation stage [9], have been identified to be the main contributors to the unpleasant stale note [10]. Furthermore, methoxybenzenes could account for 33.58% of the total volatile content in Pu-erh tea, and particularly, 1,2,3-trimethoxybenzene contributed 17.16% to the total volatiles [11]. However, no literature shows information to mask the stale note from methoxybenzenes. 

It has been pointed out that the odor from one volatile could be suppressed by another component [12]. In addition, food aromas could be improved by adding relevant compounds either to enhance a pleasant note or to decrease an unpleasant flavor due to the synergistic interaction of the volatile chemicals. For example, Xiao et al. demonstrated that the addition of ethyl octanoate, ethyl tetradecanoate, and citronellyl acetate significantly enhanced the floral note of rose essential oil, whereas the addition of geranyl acetate decreased the floral note based on sensory evaluation, olfactory threshold (OT), and Feller’s additive model analyses [13]. Lytra et al. reported that the addition of ethyl esters and acetates increased the fruity note of red wine based on OT analysis [14]. Cameleyre et al. found that the addition of 3-methylbutan-1-ol and butan-1-ol led to a significant raise in fruity note in red wine, whereas the addition of alcohols decreased the intensity through sensory evaluation and OT analysis [15]. The above studies suggest that the stale note of instant ripened Pu-erh tea products might be masked based on investigating interaction between the contributors of stale and the other notes.

It has been found that the masking effects of (*E*)-2-hexenal (green note) with 2,5-dimethylpyrazine and methional could promote thresholds of roasted note from 2,5-dimethylpyrazine by 138% and sulphur note from methional by 169% in the Oolong tea infusion [16]. Furthermore, when the mixed solutions were respectively composed of sub-threshold concentration of 4-hexanolide and some other volatiles (e.g., (*E*)-2-hexenyl hexanoate, (*Z*)-3-hexenol and indole), which was shown that the three solutions could give a noticeable astringent and heavy odor intensities on the basis of sensory evaluation [17], indicating synergistic interaction could happen among compounds even at sub-threshold concentrations. Despite of these progresses, it is still unknown which volatile has suppressive interaction with the stale note from methoxybenzenes in instant ripened Pu-erh tea products.

In this context, this study aimed to elucidate volatile chemicals in instant ripened Pu-erh tea that could mask the stale note by sensory evaluation, GC-MS, GC-O, and suppressive interaction analysis. This study could facilitate to develop processes to mask the stale note of instant ripened Pu-erh tea products.

## 2. Results and Discussion

### 2.1. Sensory Evaluation of the Aroma Profile

**Figure 1:**
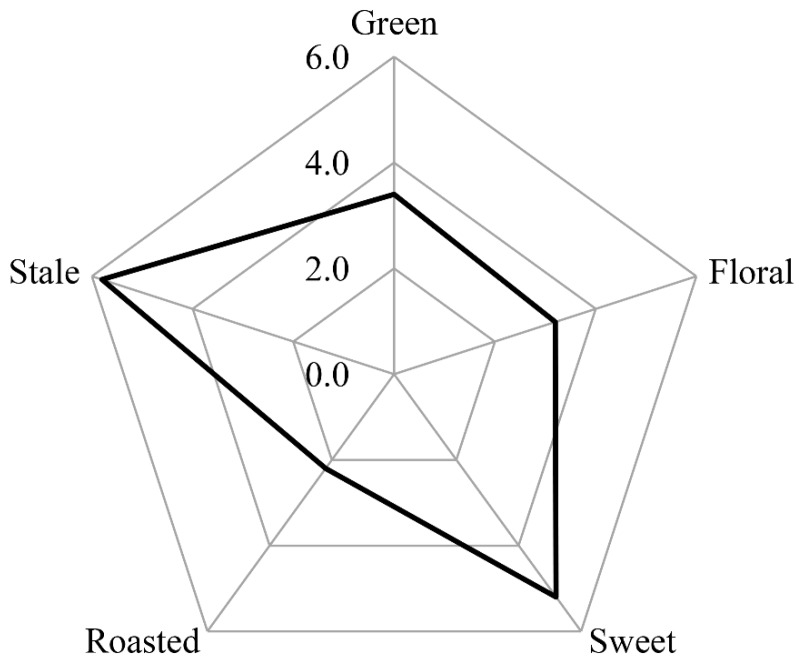
A radar map of sensory evaluation in the instant ripened Pu-erh tea.

Sensory evaluation involving the assessments of the organoleptic attributes of a product by the senses (ISO 5492) and sensory evaluation criteria has been extensively used to characterize aromatic products [18]. In this work, sensory evaluation showed that the volatile extract of instant ripened Pu-erh tea was dominated by strong stale and sweet notes, noticeable green and floral note, as well as a weak roasted note (Figure 1). This result was similar to previous studies on the aroma profile of Pu-erh teas, which showed that Pu-erh teas had a noticeable stale note [11,19].

### 2.2. GC-MS Analysis of Volatile Constituents

In order to elucidate the main volatile constituents in the SDE extract, the volatile extract was submitted to GC-MS analysis. Total 32 volatiles were detected (Table 1). Among these, 27 volatiles were identified via matching the RI and MS with those of standard chemical references, and the other five volatiles were temporarily identified by matching the RI and MS to those from the database (NIST08, NIST08s, FFNSC1.3) and references (Table 1). The volatiles with standards were quantitated using calibration curves of the standards in selective ion monitoring (SIM) mode, and the others (lacking standards) were quantified using the calibration curve of the internal reference cyclohexanone (Table 1). As a result, 1,2,3-trimethoxybenzene (260.53 μg/mL), dihydroactinidiolide (188.04 μg/mL), 1,2-dimethoxybenzene (15.54 μg/mL), <*n*->hexadecanoic acid (7.20 μg/mL), 3,4-dimethoxytoluene (6.49 μg/mL), 1,2,3-trimethoxy-5-methyl-benzene (5.32 μg/mL), 1,2,4-trimethoxybenzene (4.86 μg/mL), linalool oxide IV (4.58 μg/mL), benzyl alcohol (2.16 μg/mL), 1,2,3,4-tetramethoxybenzene (2.10 μg/mL), menthol (2.00 μg/mL), linalool (1.44 μg/mL), and linalool oxide III (1.55 μg/mL) were the dominated constitutions in the concentration (Table 1). The results were consistent with previous findings that methoxybenzenes and alcohols were the main volatile constituents in ripened Pu-erh tea products [2,8,11]. 

### 2.3. GC-O Analysis of Aroma-Active Volatiles

For investigating the volatiles that have sniffable effects on the aroma, the SDE extract of instant ripened Pu-erh tea was submitted to GC-O analysis. The results revealed that 24 volatiles had been sniffed with FD over 1, indicating that these chemicals contributed noticeably to the aroma of instant ripened Pu-erh tea (Table 2). Among these, nine chemicals, i.e., benzeneacetaldehyde (green note), linalool oxide II (sweet note), linalool (floral note), linalool oxide III (sweet note), linalool oxide IV (sweet note), *trans*-β-ionone (floral note), 1,2,3-trimethoxybenzene (stale note), 1,2,4-trimethoxybenzene (stale note), and 1,2,3-trimethoxy-5-methyl-benzene (stale note) were detected to have FD of 16 (Table 2), which were much greater than FD of other volatiles, indicating the nine chemicals (methoxybenzenes, linalool, linalool oxides, benzeneacetaldehyde, and *trans*-β-ionone) were the dominated contributors to stale, sweet, floral, and green note in instant ripened Pu-erh tea extract. Previously, alcohols (floral note), methoxybenzenes (stale/musty note), and ketones (woody/floral note) were shown to play vital roles in the special flavor of ripened Pu-erh tea [10]. In addition, 1,2,3-trimethoxybenzene and other methoxybenzenes were reported to have a strong stale/musty odor [10,11]; β-ionone was confirmed to offer a complex fruity and floral note [20]. By comparison, the main aroma contributors in instant Pu-erh tea elucidated by GC-O analysis was consistent with those of Pu-erh teas from previous studies [11].

### 2.4. Investigation of the Suppressive Interaction between the Stale Note and Other Notes

**Figure 2:**
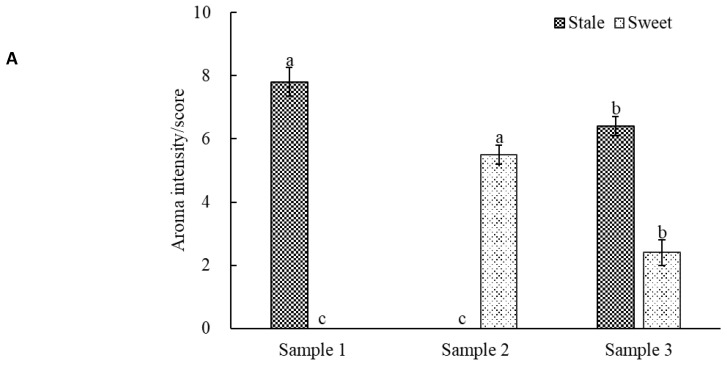

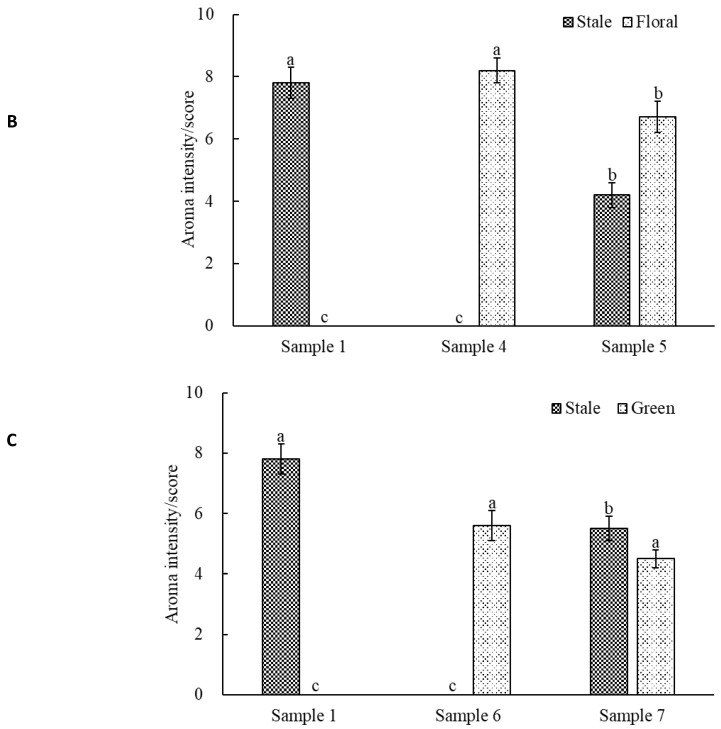
Masking test of the stale note interacting with the sweet note from linalool oxides (**A**), floral notes from linalool, *trans*-β-ionone, phenylethyl alcohol, and indole (**B**) and green note from benzeneacetaldehyde (**C**) in the instant ripened Pu-erh tea. Same aromas with different superscripts (i.e., a, b, c) have significant differences from the results (*p* < 0.05).

To investigate the suppressive interaction between the stale note and other notes (i.e., sweet, floral, and green notes), series artificial aromatic models with different odor were prepared according to the GC-O analysis, followed by sensory evaluation in their stale and other notes. In comparison to the models of stale note (sample 1#, the methoxybenzenes solution) and sweet note (sample 2#, the linalool oxides solution), the suppressive interaction model of stale and sweet notes (sample 3#, the mixture of methoxybenzenes and linalool oxides solutions) showed significant decreases in both of stale note (from 7.8 to 6.4) and sweet note (from 5.5 to 2.4), respectively (Figure 2A). By comparing the models of stale note (sample 1#, the methoxybenzenes solution) and floral note (sample 4#, the mixture of linalool, *trans*-β-ionone, phenylethyl alcohol, and indole solution), the suppressive interaction model of stale and floral notes (sample 5#, the mixture of methoxybenzenes, linalool, *trans*-β-ionone, phenylethyl alcohol, and indole solutions) showed significant decreases in stale note (from 7.8 to 4.2) and floral note (from 8.2 to 6.7) (Figure 2B). Comparing the models of stale note (sample 1#, the methoxybenzenes solution) and green note (sample 6#, the benzeneacetaldehyde solution), the suppressive interaction model of stale and green notes (sample 7#, the mixture of methoxybenzenes and benzeneacetaldehyde) showed reduced intensities in both stale (from 7.8 to 5.5) and green (from 5.6 to 4.5) notes (Figure 2C). Masking effects were reported in mixtures composed of chemicals with great difference in structure [16]. For example, the addition of (*E*)-2-hexenal (green note) masked/reduced the intensities of roasted and sulfur notes of tea infusion [16]; isoamyl acetate (fruity note) masked the stale note in fresh beer [21]; and the woody and fruity notes in wine reciprocally masked each other [22]. Our study indicated that there were a reciprocally masking effect between the stale note (methoxybenzenes) and the sweet note (linalool oxides), floral note (linalool, *trans*-β-ionone, phenylethyl alcohol, and indole) and green note (benzeneacetaldehyde), which was consistent with a previous study. 

### 2.5. Validation of Masking the Stale Note in Instant Ripened Pu-Erh Tea Infusion

**Figure 3:**
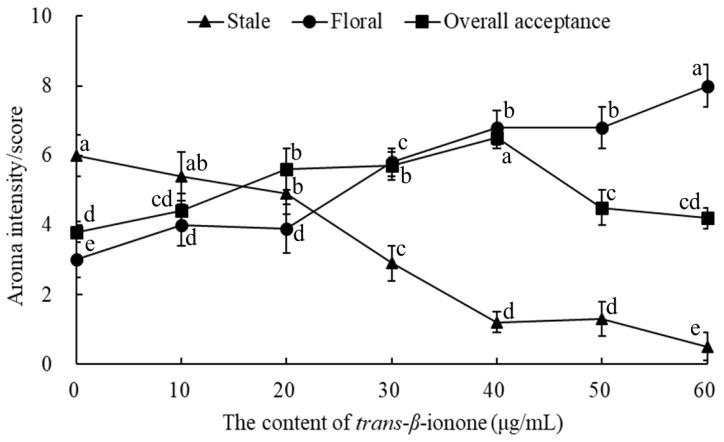
Curves of aroma intensity/score of stale and floral notes and overall acceptance with the addition of *trans*-β-ionone in the instant ripened Pu-erh tea infusion. Same aromas with different superscripts (i.e., a, b, c, d, e) have significant differences from the results (*p* < 0.05).

The above experiments showed that sweet note, floral note, and grass note could mask the stale note. In addition, it has been reported that linalool oxides (sweet note), linalool (floral note), *trans*-β-ionone (floral note), phenylethyl alcohol (floral note), indole (floral note), and benzeneacetaldehyde (green note) had thresholds of 6 [23], 6 [23], 0.007 [23], 750 [23], 140 [24], and 4 μg/L [24], respectively. Obviously, *trans*-β-ionone had the minimum threshold among the volatiles that had the reciprocally masking effect on the stale note from methoxybenzenes. Therefore, *trans*-β-ionone was added in the instant ripened Pu-erh tea infusion to validate the masking of the stale note. The result (stale and floral notes, as well the overall acceptance) as illustrated in Figure 3, the more the concentration of *trans*-β-ionone was added, the stronger intensity of the floral note and the weaker the stale note (Figure 3). When *trans*-β-ionone attained 60 μg/mL, the instant ripened Pu-erh tea infusion was hardly to sniff the stale note, which indicated that stale note can be effectively masked by the addition of *trans*-β-ionone. In addition, the overall acceptance kept on increasing along with the increase of *trans*-β-ionone concentration within 10–40 μg/mL; and addition of 40 μg/mL of *trans*-β-ionone gave the instant ripened Pu-erh tea infusion with the best overall aroma acceptance. In short, the present study indicated that an effective approach to improve the aromatic quality of instant ripened Pu-erh tea products is by masking the stale note that inherently exists in ripened Pu-erh tea products [11,19]. In addition, pulsed electric field processing (PEF) and high pressure processing (HPP) results in significant changes of volatile compounds in different lamb meat cuts [25]. Additionally, the suppression of odor intensity in volatile mixtures has been shown to occur at the neuroreceptor level [26] and is mediated by inhibitory connections in the odor maps of the olfactory bulb [14]. Furthermore, the suppressor could interact with the volatile molecule or its olfactory receptors [27]. In future, a further in-depth study is suggested to investigate how the aroma receptor binds with aroma-active chemicals that contributed to the stale and floral notes. In addition, novel processing technologies such as PEF and HPP may help to improve the aroma.

## 3. Materials and Methods

### 3.1. Instant Ripened Pu-Erh Tea

Instant ripened Pu-erh tea was processed using a combined procedure consisting of countercurrent extraction at 90 °C for 30 min, ultrafiltration, reverse osmosis concentration at 40 °C to 8–12 brix, freeze, and drying at 95 °C at Fujian DaMin Development Company (Zhangzhou, Fujian, China) in September 2016. One kilogram of the Pu-erh tea yielded approximately 400 g of instant tea.

### 3.2. Chemical Standards and Reagents

The standards 2-hexanone, benzeneacetaldehyde, linalool, linalool oxides, phenylethyl alcohol, 4-oxoisophorone, menthol, *α*-terpineol, safranal, geraniol, indole, and 2-ethyl-3-methylpyrazine were purchased from Sigma-Aldrich Co., Ltd. (St. Louis, MO, USA). The standards, 1-ethylpyrrole, benzyl alcohol, 1,2-dimethoxybenzene, 3,4-dimethoxytoluene, 1,2,3-trimethoxybenzene, 1,2,4-trimethoxybenzene, *trans*-β-ionone, 2,4-ditert- butylphenol, and dihydroactinidiolide were obtained from Alfa Aesar Co., Ltd. (Heysham, Lancashire, UK). A standard series of C_8_–C_20_ alkanes were used for retention index (RI) determination, and the internal standard cyclohexanone were purchased from Sigma-Aldrich Co., Ltd. (St. Louis, Mo, USA). Other reagents were all of analytical grade and obtained from Sinopharm Chemical Reagent Co., Ltd. (Shanghai, China).

### 3.3. Extraction of Volatiles from Instant Ripened Pu-Erh Tea

Thirty grams of Pu-erh instant tea was immersed with 300 mL of distilled water in a 500 mL flask; and 100 mL of extraction solvent (*n*-hexane) was put in the other flask of the extractor. Both flasks were put in a Likens-Nickerson apparatus and heated up to their respective boiling points. After the two flasks started to reflux, the distillation–extraction was continued for 1.5 h to allow the volatiles to be collected in the organic phase. The resultant extract was collected at room temperature and dried over anhydrous sodium sulfate overnight, followed by concentration to approximately 0.5 mL using a gentle stream of high-purity nitrogen. The concentrated extract was adjusted to the volume of 1.5 mL with *n*-hexane and stored at −20 °C temporarily before analysis.

### 3.4. Sensory Evaluation of the Aroma Profile

An aliquot of 20 μL of the volatile extract was diluted with 980 μL of ethanol (by activated carbon). Thereafter, 50 μL of the dilution sample was added onto a fragrance test strip, which was subsequently dried in the open air for 120 s prior to sensory evaluation. Sensory evaluation was conducted at room temperature under clean air conditions by 11 panelists, including five men and six women ranging from 20 to 30 years old, that were trained in odor recognition for 50 h over two months. Before sensory evaluation and, as necessary, during the session, the different concentration standard solutions of benzeneacetaldehyde, linalool, linalool oxide, 2-ethyl-3-methylpyrazine, 1,2,4-trimethoxybenzene diluted with ethanol (by activated carbon) were used to instruct the panelists to be familiar with the green, floral, sweet, roasted and stale notes, and their aroma intensities (Table 3). The panelists were asked in a random order to rate the extracts for green, floral, sweet, roasted and stale notes, and gave a score within 0–9 according to relevant references and ISO 8589 [28,29], in which zero indicates an unperceived attribute intensity and nine indicates a very strong attribute intensity. After each sniff, an interval gap of 20 s in fresh air was used refresh the olfactory fatigue, which was sufficient between individual odor assessments.

### 3.5. GC-MS Analysis of the Volatile Constituents

Nine hundred and ninety µL of the volatile extract was added with 10 µL of the internal standard cyclohexanone (1250 µg/mL), and 1 µL of the mixed solution was injected into the QP 2010 GC-MS instrument (Shimadzu, Kyoto, Japan) for analysis using a Rtx-5MS (60 m × 0.32 mm × 0.25 µm) column (Restek Corporation, Bellefonte, PA, USA). Helium was used as the carrier gas at a flow rate of 3 mL/min. The oven temperature was initially programmed at 50 °C for 2 min, then increased from 50 to 200 °C at 3 °C/min, and held at this temperature for 1 min. The temperatures of the ion source and the interface were set at 220 and 250 °C, respectively. The MS was operated in the positive electron ionization mode at 70 eV, and the MS spectra were recorded within an *m/z* range from 35 to 500 amu.

Most of the compounds were identified by matching their MS spectra and RIs on the Rtx-5MS column to those of standards. The other volatiles that lacked standards were temporarily identified by matching their MS spectra and RIs to those in the mass spectral library (NIST08, NIST08s, FFNSC1.3) and their RI values to those from relevant references. The volatiles with matching standards were quantified according to their respective calibration curves. The concentrations of the other volatiles were estimated using the calibration curve of the internal reference cyclohexanone in scan mode. 

### 3.6. GC-O Analysis of the Aroma-Active Volatiles 

GC-O analysis was performed on an Agilent 5975C-7890A GC-MS (Palo Alto, CA, USA) with a Gerstel ODP-2 olfactory detection port (Gerstel AG Enterprise, Mülheim an der Ruhr, Germany). Samples were separated and evaluated using an HP-INNOWax (60 m × 0.25 mm × 0.25 μm) column (Agilent, Palo Alto, CA, USA). The temperature at the injector port was 250 °C. A 1 μL sample was injected into the GC-O system in a splitless mode. The oven temperature was programmed as follows: An initial temperature of 40 °C was kept for 1 min and then increased to 230 °C at a speed of 5 °C/min, then held at this temperature for 3 min. Nitrogen was used as the carrier gas at a flow rate of 1.8 mL/min. 

Aroma extract dilution analysis (AEDA) was used to determine the respective notes and intensities of volatiles. A series of 4-fold dilutions (i.e., 4^0^, 4^1^, 4^2^) of the volatile extracts were prepared using the solvent *n*-hexane. The sniffing test was performed by three well-trained panelists in an alternating order at 2 h intervals with reference compounds. All panelists were trained for 60 h over a period of one month. Upon sniffing the effluents from the sniffing mask, the panelists recorded the retention time and aroma descriptors. The odor intensity of fragrance chemicals was evaluated with flavor dilution (FD) factors.

### 3.7. Investigation of the Suppressive Interaction between the Stale Note and Other Notes

Seven aromatic models were prepared artificially with volatiles and deodorized ethanol (by activated carbon) to a final volume of 5.0 mL, according to a previous method with minor modification [30] (Table 4). Volatiles in the samples were prepared with concentrations the same as those detected in instant ripened Pu-erh tea. All samples were evaluated in the aroma intensities by using the method described in Section “Sensory evaluation of the aroma profile”.

### 3.8. Validation of Masking the Stale Note in Instant Ripened Pu-Erh Tea Infusion

Two grams of instant ripened Pu-erh tea powder was dissolved in 100 mL water at 80 °C [16]. The instant ripened Pu-erh tea infusion was added with *trans*-β-ionone that showed the strongest suppression effect on stale note, at series concentrations (i.e., 0, 10, 20, 30, 40, 50, and 60 μg/mL, respectively). Thereafter, all the samples were sensory evaluated in stale and floral notes by using the method described in Section “Sensory evaluation of the aroma profile”. In addition to this, the overall acceptance was used to evaluate how we feel the aroma. During the evaluation, a 0–9 scale system was used, with zero points indicating poor overall acceptance, and nine points indicating good overall acceptance.

### 3.9. Statistical Analysis

All experiments were repeated three times. Mean values and standard deviations of sensory evaluation and quantitative analysis were calculated using the SPSS-IBM 19.0 software (IBM company, Chicago, IL, USA) and Microsoft Excel 2013. The analysis of panelist effect and removal of outliers by normal distribution were performed using the SPSS-IBM 19.0 software. Analysis of variance (ANOVA) was conducted via Duncan’s multiple comparison tests (*p* < 0.05) using the SPSS-IBM 19.0 software [31].

## 4. Conclusions

In summary, the stale note from methoxybenzenes had a reciprocal masking interaction with sweet, floral, and green notes, respectively. The validation experiment showed that *trans*-β-ionone significantly eliminated stale note and improved the overall aromatic acceptance of instant ripened Pu-erh tea infusion. These results indicate that the masking interaction could be applied to mask the stale note, providing an effective approach to mask the stale note of instant ripened Pu-erh tea. In the future, the study might focus on the mechanism of interaction between aroma compounds and how they bind to olfactory receptors and explore novel processing technologies to improve the aromatic components. 

## Figures and Tables

**Table 1:** Identification and quantitative analysis of the volatiles from instant ripened Pu-erh tea.

No.	Volatiles	Rtx-5MS		Characteristic Ion Fragment	Std ^c^	Calibration Equation ^d^	Range (μg/mL)	*R* ^2^	CF ^e^	Concentration (μg/mL)
		^a^ RI^1^	^b^ RI^2^							
Aldehydes										
1	Benzeneacetaldehyde	1045	1045	91 92 120	MS, Std	*Y* = 1.39737*X* − 0.05430	0.025–5	0.9995	0.719	0.83 ± 0.04
2	Safranal	1203	1203	107 91 121	MS, Std	*Y* = 2.02835*X* − 0.25929	0.025–5	0.9991	0.504	0.13 ± 0.01
3	1-ethyl-1*H*-Pyrrole-2-carboxaldehyde	1051	1052	39 94 123	MS	c	-	-	-	0.70 ± 0.01
Alcohols										
4	Benzyl alcohol	1037	1037	108 79 107	MS, Std	*Y* = 0.19632*X* − 0.43109	0.025–5	0.9970	0.541	2.16 ± 0.0.11
5	Linalool oxide I	1074	1074	59 94 43	MS, Std	*Y* = 4.30922*X* − 0.07267	0.025–5	0.9997	0.233	0.67 ± 0.01
6	Linalool oxide II	1090	1088	59 43 94	MS, Std	*Y* = 2.84204*X* − 0.06826	0.025–5	0.9997	0.354	0.79 ± 0.01
7	Linalool	1102	1101	71 41 93	MS, Std	*Y* = 2.29175*X* − 0.17466	0.025–5	0.9996	0.719	1.44 ± 0.03
8	Hotrienol	1107	1104	71 82 152	MS	c	-	-	-	0.27 ± 0.01
9	3-Octen-2-ol	1110	1114	43 71	MS, Std	*Y* = 3.63218*X* − 0.20835	0.025–5	0.9991	0.277	0.73 ± 0.01
10	Phenylethyl alcohol	1116	1116	91 92 122	MS, Std	*Y* = 7.61778*X* − 0.53450	0.025–5	0.9995	0.132	0.21 ± 0.01
11	Linalool oxide III *	1172	1173	43 94 67	MS	*Y* = 2.84204*X* − 0.06826	0.025–5	0.9997	0.354	1.55 ± 0.03
12	Menthol	1176	1178	71 81 95	MS, Std	*Y* = 0.24437*X* − 0.01346	0.025–5	0.9996	4.122	2.00 ± 0.17
13	Linalool oxide IV *	1178	1175	43 94 67	MS	*Y* = 2.84204*X* − 0.06826	0.025–5	0.9997	0.354	4.58 ± 0.04
14	α-Terpineol	1193	1195	59 93 121	MS, Std	*Y* = 1.24692*X* − 0.07854	0.025–5	0.9994	0.808	1.17 ± 0.01
15	Nerol	1232	1232	69 41 93	MS, Std	*Y* = 2.02835*X* − *0.25929*	0.025–5	0.9992	0.413	0.24 ± 0.01
16	Syringol	1249	1239	93 139 154	MS	c	-	-	-	0.59 ± 0.02
17	Geraniol	1259	1259	69 41 68	MS, Std	*Y* = 1.66627*X* − 0.18129	0.025–5	0.9994	0.603	0.78 ± 0.02
18	2,4-Ditert-butylphenol	1518	1513	191 57 206	MS, Std	*Y* = 17.5470*X* + 0.15020	0.005–5	0.9998	0.057	0.01 ± 0.00
Ketones										
19	2-Hexanone	-	792	43 58 57	MS, Std	*Y* = 0.24039*X* − 0.26212	0.025–5	0.9990	0.264	0.94 ± 0.04
20	4-Oxoisophorone	1146	1147	68 96 102	MS, Std	*Y* = 0.56877*X* − 0.02251	0.025–5	0.9996	1.759	0.31 ± 0.01
21	*trans*-β*-*Ionone	1491	1490	177 43 41	MS, Std	*Y* = 7.78433*X* − 0.12726	0.025–5	0.9995	0.129	0.16 ± 0.01
Esters										
22	Methyl salicylate	1197	1197	39 92 120	MS, Std	*Y* = 1.07835*X* − 0.05296	0.025–5	0.9990	2.416	0.05 ± 0.01
23	Dihydroactinidiolide	1538	1538	111 43 137	MS, Std	*Y* = 0.04390*X* − 0.07338	20–500	0.9969	2.210	188.04 ± 3.35
Methoxybenzenes										
24	1,2-Dimethoxybenzene	1151	1149	138 95 123	MS, Std	*Y* = 0.20751*X* − 0.27376	5–35	0.9976	0.430	15.54 ± 0.23
25	3,4-Dimethoxytoluene	1243	1246	152 137 109	MS, Std	*Y* = 0.16900X − 0.15431	0.05–10	0.9969	0.533	6.49 ± 0.06
26	1,2,3-Trimethoxybenzene	1321	1315	117 90 89	MS, Std	*Y* = 0.12953*X* − 0.00718	20–500	0.9997	7.774	260.53 ± 3.92
27	1,2,4-Trimethoxybenzene	1378	1378	168 103 110	MS, Std	*Y* = 2.22856*X* − 0.01142	5–35	1.0000	0.441	4.86 ± 0.12
28	1,2,3-Trimethoxy-5-methyl-benzene *	1410	1410	168 103 125	MS	*Y* = 2.22856*X* − 0.01142	5–35	1.0000	0.441	5.32 ± 0.09
29	1,2,3,4-Tetramethoxybenzene	1453	1449	97 140 198	MS	c	-	-	-	2.10 ± 0.06
Others					MS					
30	1-Ethylpyrrole	815	815	80 95 67	MS, Std	*Y* = 1.01083*X* − 0.00793	0.025–5	0.9996	0.990	0.40 ± 0.01
31	Indole	1299	1300	117 90 89	MS, Std	*Y* = 4.59138*X* − 0.47327	0.025–5	0.9994	0.221	0.26 ± 0.01
32	<*n*->Hexadecanoic acid	-	1962	73 256	MS	c	-	-	-	7.20 ± 0.08

^a^ RI^1^ was obtained by GC-MS analysis using the Rtx-5MS column. ^b^ RI^2^ was reported in the database on the web (http://webbook.nist.gov/chemistry/) and was analyzed using a column similar to Rtx-5MS. ^c^ Std indicates that the identification was confirmed by matching a standard. ^d^ All of the equations of the calibration curves of authentic standard chemicals (ASCs) were calculated in the SIM mode, where *X* is the ratio of the concentration of the ASC to that of the internal standard (IS) and *Y* is the ratio of the peak area of the ASC to that of the IS and the concentrations of volatiles that currently lack standards were estimated using the calibration curve of the internal reference of cyclohexanone in the scan mode. ^e^ CF represents correction factors using this formula: CF = (As/Ms)/(Ar/Mr), As represents the corresponding quantitative ion (SIM mode) area of the IS, Ar represents the corresponding quantitative ion (SIM mode) area of the ASC, Ms represents the concentration of IS, Mr represents the concentration of the ASC. * represents that the standard curve of linalool oxide II is used to quantify linalool oxide III and linalool oxide IV; the standard curve of 1,2,4-trimethoxybenzene is used to quantify 1,2,3-trimethoxy-5-methyl-benzene.

**Table 2:** Odor descriptions, flavor dilution factors (FD), and aroma intensity of the aroma-active compounds.

No.	^a^ RI^3^	^b^ RI^4^	Volatiles	Odor Description	FD
Aldehydes					
1	1655	1650	Benzeneacetaldehyde	Green	16
2	1204	1203	Safranal	Green	1
3	1619	1616	1-ethyl-1*H*-pyrrole-2-carboxaldehyde	Green	4
Alcohols					
4	1878	1877	Benzyl alcohol	Sweet *, roasted	4
5	1439	1435	Linalool oxide I	Sweet	4
6	1468	1470	Linalool oxide II	Sweet	16
7	1543	1549	Linalool	Floral	16
8	1915	1919	Phenylethyl alcohol	Floral	4
9	1753	1750	Linalool oxide III	Sweet	16
10	1636	1632	Menthol	Green	1
11	1780	1775	Linalool oxide IV	Sweet	16
12	1718	1715	*α*-Terpineol	Wood	4
13	-	2321	2,4-Ditert-butylphenol	Green	1
Ketones					
14	1105	1102	2-Hexanone	Fruity, floral	1
15	1955	1954	*trans*-β-Ionone	Floral	16
Esters					
16	1763	1759	Methyl salicylate	Sweet	1
Methoxybenzenes					
17	1731	1731	1,2-Dimethoxybenzene	Stale	4
18	1807	1806	3,4-Dimethoxytoluene	Stale	1
19	1961	1955	1,2,3-Trimethoxybenzene	Stale	16
20	-	2094	1,2,4-Trimethoxybenzene	Stale	16
21	-	2041	1,2,3-Trimethoxy-5-methyl-benzene	Stale	16
22	-	2321	1,2,3,4-Tetramethoxybenzene	Green	4
Others					
23	1179	1178	1-Ethylpyrrole	Roasted	4
24	-	2435	Indole	Floral	4

^a^ RI^3^ was obtained by GC-O analysis using a HP-INNOWax column. ^b^ RI^4^ was reported in the database on the web (http://webbook.nist.gov/chemistry/) and was analyzed by GC-MS analysis using a column similar to HP-INNOWax column. “*” is the main smell of olfactometry among a number of odors.

**Table 3:** Sensory evaluation criteria for instant ripened Pu-erh tea.

Aroma Characteristic	Standard Solution	Concentration for Aroma Intensity of 0 Scores (μL/L)	Concentration for Aroma Intensity of 9 Scores (μL/L)
Green	Benzeneacetaldehyde	100	900
Floral	Linalool	20	180
Sweet	Linalool oxide	10	90
Roasted	2-Ethyl-3-methylpyrazine	130	1170
Stale	1,2,4-Trimethoxybenzene	1147	10323

**Table 4:** Suppressive interaction model of aroma compounds.

Samples	Aroma Description	Compound Composition and Concentration
Sample 1#	Stale	Mixture of 15.54 μg/mL of 1,2-dimethoxybenzene, 260.53 μg/mL of 1,2,3-trimethoxybenzene and 4.86 μg/mL of 1,2,4-trimethoxybenzene
Sample 2#	Sweet	Mixture of 0.67 μg/mL of linalool oxide I and 0.79 μg/mL of linalool oxide II
Sample 3#	Stale + sweet	Mixture of samples 1# and samples 2#
Sample 4#	Floral	Mixture of 1.44 μg/mL of linalool, 0.16 μg/mL of *trans*-β-ionone, 0.21 μg/mL of phenylethyl alcohol and 0.26 μg/mL of indole
Sample 5#	Stale + floral	Mixture of samples 1# and samples 4#
Sample 6#	Green	Mixture of 0.83 μg/mL of benzeneacetaldehyde
Sample 7#	Stale + green	Mixture of samples 1# and samples 6#
